# Postoperative complications and clinical outcomes among patients undergoing thoracic and gastrointestinal cancer surgery: A prospective cohort study

**DOI:** 10.5935/0103-507X.20160012

**Published:** 2016

**Authors:** Frank Daniel Martos-Benítez, Anarelys Gutiérrez-Noyola, Adisbel Echevarría-Víctores

**Affiliations:** 1 Oncological Intensive Care Unit, Instituto de Oncología y Radiobiología - La Habana, Cuba.

**Keywords:** Gastrointestinal neoplasms/surgery, Gastrointestinal neoplasms/complications, Thoracic neoplasms/surgery, Thoracic neoplasms/complications, Postoperative complication, Hospital mortality, Length of hospital stay, Treatment outcome

## Abstract

**Objective:**

This study sought to determine the influence of postoperative complications on the clinical outcomes of patients who underwent thoracic and gastrointestinal cancer surgery.

**Methods:**

A prospective cohort study was conducted regarding 179 consecutive patients who received thorax or digestive tract surgery due to cancer and were admitted to an oncological intensive care unit. The Postoperative Morbidity Survey was used to evaluate the incidence of postoperative complications. The influence of postoperative complications on both mortality and length of hospital stay were also assessed.

**Results:**

Postoperative complications were found for 54 patients (30.2%); the most common complications were respiratory problems (14.5%), pain (12.9%), cardiovascular problems (11.7%), infectious disease (11.2%), and surgical wounds (10.1%). A multivariate logistic regression found that respiratory complications (OR = 18.68; 95%CI = 5.59 - 62.39; p < 0.0001), cardiovascular problems (OR = 5.06, 95%CI = 1.49 - 17.13; p = 0.009), gastrointestinal problems (OR = 26.09; 95%CI = 6.80 - 100.16; p < 0.0001), infectious diseases (OR = 20.55; 95%CI = 5.99 - 70.56; p < 0.0001) and renal complications (OR = 18.27; 95%CI = 3.88 - 83.35; p < 0.0001) were independently associated with hospital mortality. The occurrence of at least one complication increased the likelihood of remaining hospitalized (log-rank test, p = 0.002).

**Conclusions:**

Postoperative complications are frequent disorders that are associated with poor clinical outcomes; thus, structural and procedural changes should be implemented to reduce postoperative morbidity and mortality.

## Abstract (es)

**Objetivo:**

Determinar la influencia de las complicaciones postoperatorias sobre los resultados clínicos en pacientes operados por cáncer torácico y gastrointestinal.

**Métodos:**

Se realizó un estudio de cohorte prospectivo de 179 pacientes consecutivos que fueron operados de tórax o vías digestivas por cáncer y admitidos en una unidad de cuidados intensivos oncológicos. Se evaluó la incidencia de las complicaciones postoperatorias mediante el *Postoperative Morbidity Survey* y su influencia sobre la mortalidad y estadía hospitalaria.

**Resultados:**

Se presentaron complicaciones postoperatorias en 54 sujetos (30,2%); las más frecuentes fueron las pulmonares (14,5%), el dolor (12,9%), las cardiovasculares (11,7%), las infecciosas (11,2%) y las de la herida quirúrgica (10,1%). En el análisis multivariado de regresión logística las complicaciones pulmonares (OR 18,68; IC95% 5,59 - 62,39; p < 0,0001), cardiovasculares (OR 5,06; IC95% 1,49 - 17,13; p = 0,009), gastrointestinales (OR 26,09; IC95% 6,80 - 100,16; p < 0,0001), infecciosas (OR 20,55; IC95% 5,99 - 70,56; p < 0,0001) y renales (OR 18,27; IC95% 3,88 - 83,35; p < 0,0001) se relacionaron de forma independiente con la mortalidad hospitalaria. La ocurrencia de al menos una complicación incrementó la probabilidad de permanecer hospitalizado (Log Rank test; p = 0,002).

**Conclusiones:**

Las complicaciones postoperatorias son trastornos frecuentes y asociados con malos resultados clínicos, por lo que se deben realizar cambios estructurales y de proceso para reducir la morbilidad y mortalidad postoperatorias.

## INTRODUCTION

The incidence of cancer is increasing worldwide and becoming a major public health problem.^(1,2)^ Cancer is the leading cause of death in many countries, including Cuba.^(3)^ Gastrointestinal and lung cancers are highly prevalent in the world and are associated with high mortality rates.^(4)^ In the early stages of these cancers, surgery is the most effective treatment. Despite the benefits of surgery, however, they are not free of complications, including death.^(5)^

Postoperative complications are frequent events, particularly among patients at high risk.^(6)^ These complications have both clinical effects during the immediate postoperative period and long-term effects on quality of life impairment and increased mortality.^(5,7)^ The complications that occur after surgery are challenging for physicians because they are sometimes unpredictable, have relatively sudden onset, and can develop quickly toward death. Many patients with acute pathophysiological disorders require admission to intensive care units (ICU) for better disease control and management.

Approximately 200 million people are estimated to undergo major non-cardiac surgery each year, and nearly 1 million die as a result.^(8)^ Knowledge of the factors associated with postoperative mortality allows for better clinical decision making, not only to act and correct modifiable factors but also to operate at the right time and optimize surgical outcomes.

Although the likely predictors of death after surgery have been studied extensively,^(9-12)^ the available knowledge regarding the effect of postoperative complications on mortality is limited, especially in the context of patients who have undergone cancer surgery. Thus, the current study was conducted to determine the influence of postoperative complications on both mortality and hospital stay among patients undergoing surgery for thoracic and gastrointestinal cancer.

## METHODS

A prospective cohort study was conducted from January 2014 to December 2014 at the oncological ICU (OICU) of the Institute of Oncology and Radiobiology (Instituto de Oncología y Radiobiología; IOR) in Cuba. The IOR is a tertiary referral hospital for the care of patients with cancer, and it has 220 beds for in-patient hospitalization. The OICU has 12 beds and cares for approximately 400 patients undergoing cancer surgery, either elective or emergency, each year. The current study was conducted in accordance with the Declaration of Helsinki, and it was approved by the Scientific Council and the Ethics Committee for Scientific Research of the OICU (November 2013). Written informed consent was obtained from all patients.

**Figure 1:**
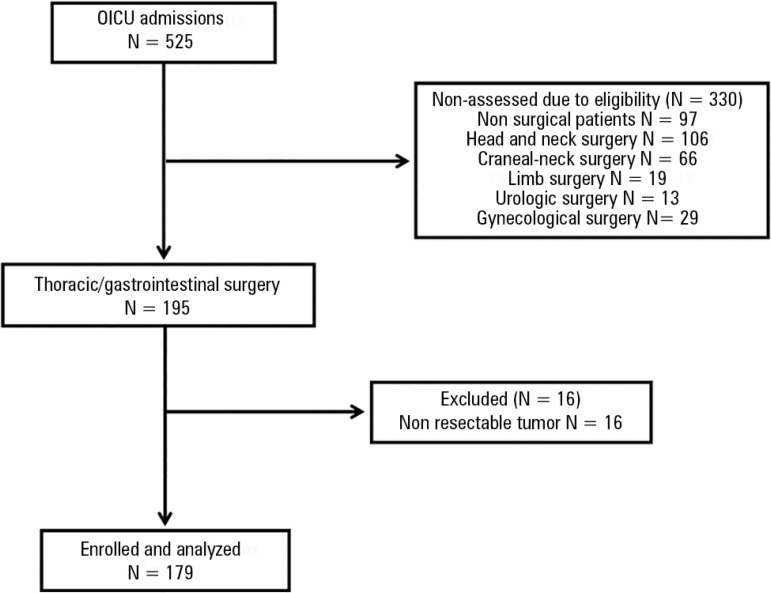
Flowchart of participants. OICU - oncological intensive care unit.

A total of 525 consecutive patients were admitted to the OICU during the study period; of these patients, 195 underwent either thoracic surgery (thoracic wall, lung, or mediastinal resection) or digestive tract surgery (esophagus, stomach, hepato-biliary-pancreatic, small intestine, or colon-rectum) for cancer. Patients undergoing palliative surgery and those for whom ≥ 75% of the tumor could not be removed (including metastases; Figure 1) were excluded because patients in advanced stages can show basic features that distinguish them from those with cancer in remission. Thus, their exclusion reduced the risk of selection bias.

The following demographic and clinical data were obtained at OICU admission: age, sex, emergency surgery, American Society of Anesthesiology (ASA) class ≥ 3,^(13)^ location of the surgery, surgical time, adverse intraoperative event,^(14)^ and Acute Physiology and Chronic Health Evaluation (APACHE) II scale score.^(15)^

Postoperative complications were monitored daily throughout the patient's stay in the OICU, and these complications were assessed using the Postoperative Morbidity Survey (POMS). The POMS measures nine domains of morbidity, in which the presence or absence of morbidity according to the defining criteria is recorded for each domain (Table 1). The POMS accurately describes the prevalence pattern of postoperative complications.^(16)^ This system has been well validated across different populations and provides objective evidence of postoperative complications.^(17,18)^

**Table 1:** Domains, definition criteria, and data sources of the Postoperative Morbidity Survey

**Type of postoperative complication**	**Defining criteria**	**Data source**
Respiratory	Need for oxygen or respiratory support	Patient monitoring, table of clinical indications
Microbiology	Antibiotics* or pyrexia > 38°C over previous 24 hours	Record of vital signs, table of clinical indications
Renal	Oliguria, raised serum creatinine levels, new urinary catheter	Record of fluid balance, analytical results, patient monitoring
Gastrointestinal	Failure of enteral feeding	Questions for the patient, record of fluid balance, table of clinical indications
Cardiovascular	Diagnosis or treatment within last 24 hours for any of the following: new acute myocardial infarct, hypotension, arrhythmia, cardiogenic pulmonary edema, or thrombotic event	Table of clinical indications, medical notes
Neurological	Cerebrovascular accident/transient ischemic attack, confusion, delirium, coma	Medical notes, questions for the patient
Hematological	Use of red cells, platelets, fresh-frozen plasma, cryoprecipitates within last 24 hours	Record of fluid balance, table of clinical indications
Surgical wound	Infection/wound dehiscence needing exploration or drainage of pus	Medical notes, microbiological results
Pain	New pain requiring parenteral opioids or additional regional analgesia	Table of clinical indications, questions for the patient

Source: Shah N, Hamilton M. Clinical review: Can we predict which patients are at risk of complications following surgery? Crit Care. 2013;17(3):226.

* Different from that used prophylactically.

The assessed clinical outcomes were mortality in the OICU, length of OICU stay, hospital mortality, and length of hospital stay. Hospital mortality was the primary response variable analyzed.

### Statistical analyses

Categorical variables are shown as percentages, whereas continuous variables are represented as means and standard deviations (SD) or medians with interquartile ranges (IQR) depending on whether the population was normally distributed. Between-group comparisons were performed using the chi-square (χ^2^) test or Fisher's exact test based on which test was more suitable for qualitative variables. For continuous variables, *t*-tests or nonparametric procedures (e.g., the Mann-Whitney *U* test or a Kruskal-Wallis one-way ANOVA) were used depending on whether the population was normally distributed. The Kaplan-Meier method was used to assess the probability that patients had to remain hospitalized.

The primary statistical analyses were performed for hospital mortality using multivariate binary logistic regression models. Continuous variables without normal distributions were transformed before being introduced into the models. No parsimonious models were used because the analyses were associative rather than predictive. However, the number of confounds in the analyses were decreased to reduce the complexity of the models. This reduction was achieved over two phases: 1) Only those variables with a p-value of ≤ 0.25 in the univariate analysis and obvious clinical implications (e.g., emergency surgery and age were not included as isolated variables because both were considered to calculate the APACHE II score) were included; and 2) only variables with strong effects on the estimates were selected via *backward elimination using the likelihood ratio* (those with p-values ≤ 0.25 were retained, and those with p-values ≥ 0.30 were dropped). Then, the models for each postoperative POMS complication were built. The goodness of fit of the models was evaluated using the Hosmer-Lemeshow test, where a p-value of ≥ 0.05 indicates a good fit. The results are shown as odds ratios (ORs) with 95% confidence intervals (CI).

Hypothesis tests showing a two-tailed p-value of ≤ 0.05 were considered as significant. IBM^®^ SPSS^®^ 20.0 (IBM, Armonk, NY, USA) was used for all analyses.

## RESULTS

Exactly 179 patients were assessed (Figure 1), and their general features are shown in table 2. Gastrointestinal surgeries were more frequent than thoracic surgeries (63.1% *versus* 36.9%). The most common gastrointestinal surgery was colorectal (62.0%), whereas lung resection (84.9%) was the predominant thoracic surgery. The risk of death on admission to the OICU was low according to the APACHE II scale, with a median of 11.1% (IQR = 8.1% - 14.6%), although 24% of patients had a risk of death ≥ 20%. Invasive respiratory support was necessary for 14 patients (7.8%), and 10 patients (5.6%) required vasoactive drugs. Six patients (3.4%) were re-admitted to the OICU during the same hospitalization.

**Table 2:** General patient characteristics

**Features**	**N = 179**
Age (years)	63.0 (54.0-70.0)
Age ≥ 65 (years)	77 (43.5)
Gender (male)	93 (52.0)
ASA Class ≥ 3	17 (9.5)
Surgical localization	
Thorax	66 (36.9)
Lung resection	56 (31.3)
Thoracic wall	3 (1.7)
Mediastinum	7 (3.9)
Gastrointestinal	113 (63.1)
Esophagus	4 (2.2)
Stomach	31 (17.3)
Small intestine	4 (2.2)
Hepatic/Biliary/Pancreatic	4 (2.2)
Colorectal	70 (39.1)
Emergency surgery	17 (9.5)
Intraoperative events	14 (7.8)
Surgical time (minutes)	246.3 (54.6)
APACHE II scale (score)	10.1 (8.1-12.0)
APACHE II scale ≥ 15 (score)	43 (24.0)

ASA - American Society of Anesthesiology; APACHE - Acute Physiology and Chronic Health Evaluation. The results are shown as medians (IQR), numbers and percentages, and means with standard deviations.

At least one postoperative complication occurred across 54 participants (30.2%) for a total of 151 complications; 23 patients (12.8%) had more than one complication. As shown in table 3, postoperative pain was significantly more frequent among patients undergoing thoracic surgery, whereas gastrointestinal complications were significantly more frequent among patients undergoing digestive tract surgery. The rates of other types of complications did not differ between groups.

**Table 3:** Postoperative complications by surgical location

**Complications**	**Total**	**Gastrointestinal surgery**	**Thoracic surgery**	**p value**
	**N = 179**	**N = 113**	**N = 66**	
Respiratory	26 (14.5)	14 (12.4)	12 (18.2)	0.400
Cardiovascular	21 (11.7)	12 (10.6)	9 (13.6)	0.716
Gastrointestinal	16 (8.9)	15 (13.3)	1 (1.5)	0.017
Neurological	12 (6.7)	10 (8.8)	2 (3.0)	0.215
Hematological	5 (2.8)	2 (1.8)	3 (4.5)	0.359
Infectious	20 (11.2)	16 (14.2)	4 (6.1)	0.158
Renal	10 (5.6)	8 (7.1)	2 (3.0)	0.279
Surgical wound	18 (10.1)	14 (12.4)	4 (6.1)	0.272
Pain	23 (12.9)	6 (5.3)	17 (25.8)	< 0.001

Results are shown as numbers and percentages.

No significant differences were found among patients undergoing thoracic or digestive tract surgery with regard to the occurrence of at least one complication (31.8% thorax *versus* 29.2% gastrointestinal; p = 0.842) or more than one complication (9.1% thorax *versus* 15.0% gastrointestinal; p = 0.359). Although the median number of complications was lower among patients undergoing thoracic surgery than those undergoing digestive tract surgery (1.0 [IQR = 1.0 - 2.0] *versus* 2.0 [IQR 1.0 - 3.0]), this difference was not significant (p = 0.073).

Emergency surgeries were not associated with the development of postoperative complications compared with elective surgeries (16.7% emergency *versus* 6.4% elective; p = 0.061). In turn, the occurrence of any intraoperative event was significantly associated with an increased risk of developing complications (16.7% *versus* 4.0%; p = 0.012). Similarly, the median APACHE II score on admission to the OICU was significantly higher among patients who developed a postoperative complication compared with those who did not have complications (12.1 [IQR = 9.9 - 17.3] *versus* 9.9 [IQR = 7.7 - 14.6]; p = 0.005).

Thirteen patients (7.3%) died during their stay at the OICU. Mortality at the OICU was significantly higher among individuals who developed at least one complication compared with those without any postoperative complications (22.2% *versus* 0.8%; p < 0.001).

Moreover, the overall hospital mortality was 10.1%. As shown in table 4, the preoperative and intraoperative factors associated with hospital mortality in the univariate analysis were age ≥ 65 years, emergency surgery, intraoperative events, and the APACHE II scale score on OICU admission.

**Table 4:** Preoperative and intraoperative factors associated with hospital mortality

**Variables**	**Deaths**	**Alive**	**p value**
	**N = 18**	**N = 161**	
Age (years)	67 (53.0-73.8)	62 (54-70)	0.152
Age ≥ 65 (years)	13 (72.2)	64 (40.3)	0.019
Gender (male)	11 (61.1)	82 (50.9)	0.568
ASA class ≥ 3	4 (22.2)	13 (8.1)	0.091
Surgical localization			0.106
Thorax	3 (16.7)	63 (39.1)	
Gastrointestinal	15 (83.3)	98 (60.9)	
Emergency surgery	6 (33.3)	11 (6.8)	0.003
Intraoperative events	5 (27.8)	9 (5.6)	0.007
Surgical time (minutes)	249.1 (60.3)	238.8 (42.4)	0.489
APACHE II scale (score)	15 (10.7-21.6)	10.4 (7.9-14.6)	0.003
APACHE II scale ≥ 15 (score)	9 (50.0)	34 (21.1)	0.016

ASA - American Society of Anesthesiology; APACHE - Acute Physiology and Chronic Health Evaluation. The results are shown as medians (IQR), numbers and percentages, and means with standard deviations.

Lung, cardiovascular, gastrointestinal, neurological, infectious disease, and renal complications were significantly more frequent among the patients who died in hospital compared with those discharged from the hospital according to the univariate analysis (Table 5). After adjusting for covariates, the multivariate logistic regression models found that the same types of complications (excluding neurological complications) were independent risk factors for hospital death (Table 5). Table 5 also shows that all of the models used to assess the influence of postoperative complications on hospital mortality showed significant goodness of fit according to the Hosmer-Lemeshow test (p-value ≥ 0.05).

**Table 5:** The influence of postoperative complications on hospital mortality

**Complication**	**Univariate analysis**			Multivariate analysis*		
	**Deaths**	**Living**	**p value**	**OR†**	**p value**	**Hosmer-Lemeshow**
	**N = 18**	**N = 161**		**(95%CI)**		**(χ^2^; p value)**
Respiratory	12 (66.7)	14 (8.7)	< 0.0001	18.68 (5.59 - 62.39)	< 0.0001	7.31; 0.504
Cardiovascular	6 (33.3)	15 (9.3)	0.009	5.06 (1.49 - 17.13)	0.009	7.64; 0.469
Gastrointestinal	10 (55.6)	6 (3.7)	< 0.0001	26.09 (6.80 - 100.16)	< 0.0001	6.50; 0.591
Neurological	5 (27.8)	7 (4.3)	0.003	3.66 (0.86 - 15.60)	0.079	6.96; 0.541
Hematological	1 (5.6)	4 (2.5)	0.415	2.76 (0.28 - 27.60)	0.389	4.72; 0.787
Infectious	11 (61.1)	9 (5.6)	< 0.0001	20.55 (5.99 - 70.56)	< 0.0001	4.30; 0.829
Renal	6 (33.3)	4 (2.5)	< 0.0001	18.27 (3.88 - 83.35)	< 0.0001	7.36; 0.492
Surgical wound	4 (22.2)	14 (8.7)	0.111	2.15 (0.40 - 10.15)	0.223	4.55; 0.809
Pain	5 (27.8)	18 (11.2)	0.076	2.31 (0.54 - 12.32)	0.296	4.63; 0.799

OR - odds ratio; CI - confidence interval.

* Multivariate logistic regression analysis. † Adjusted for intraoperative events and APACHE II score on hospitalization at UCIO. The results are shown as numbers and percentages.

† Adjusted for intraoperative events and APACHE II score on hospitalization at UCIO. The results are shown as numbers and percentages.

The median OICU stay was 3.0 days (IQR = 3.0 - 5.0 days), whereas the median hospital stay was 8.0 days (IQR = 7.0 - 11.0 days). The OICU stay was significantly longer for patients who presented with postoperative complications than those without postoperative complications (median = 5.0 days [IQR = 3.0 - 10.25 days] *versus* 3.0 days [IQR = 2.0 - 4.0 days]; p < 0.001).

**Figure 2:**
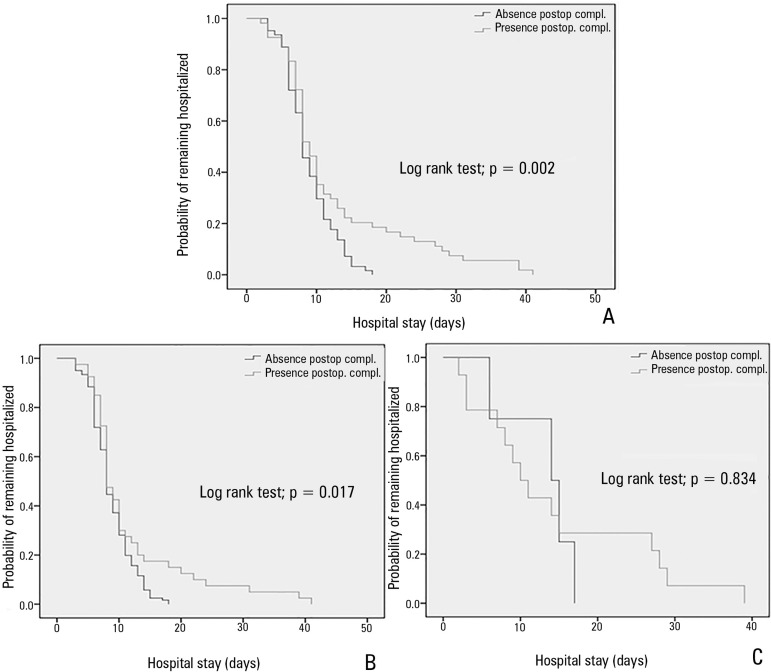
Kaplan-Meier curves for hospital stay depending on the presence of postoperative complications. A) all patients; B) discharged patients; and C) patient deaths. Postop. compl. - postoperative complications.

Moreover, significant differences were not found with regard to the hospital stays between patients with and without postoperative complications (median of complications = 9.0 days [IQR = 7.0 - 14.0 days] *versus* no complications = 8.0 days [IQR = 6.0 - 11.0 days]; p = 0.096). The Kaplan-Meier analysis showed that patients who presented with complications were more likely to stay in the hospital; this finding was particularly salient after the 10^th^ day in the hospital (Figure 2A). The same finding was true for the group of discharged patients (Figure 2B).

## DISCUSSION

Patients who undergo surgery are a particularly sensitive population because of the occurrence of complications that have great psychological and emotional effects. Furthermore, these situations demand extra effort and coordinated work among the healthcare staff as well as additional hospital costs.^(19)^

Thoracic and gastrointestinal surgeries are the most common procedures among patients with cancer. The current study assessed the influence of postoperative complications on hospital mortality and length of stay. This research has the strength of being prospective, and the evaluation of postoperative complications was performed using the POMS, which was previously used for different scenarios.^(17,18)^ Both of these elements minimize sources of bias. The sample size and the fact that the study was conducted at a single specialized center might limit the generalization of the results.

The major findings of this study are that respiratory, cardiovascular, gastrointestinal, infectious and renal complications are independently associated with hospital mortality. Another significant result was that the presence of at least one complication was associated with a greater likelihood of staying in the hospital.

Mortality and prolonged postoperative stays are most likely necessarily associated with the development of complications; however, the magnitude of this effect might vary depending on the type of complication, a possibility that we aimed to prove in this study. Other authors have previously noted the association between postoperative complications and adverse clinical outcomes. Borja-Cacho et al.^(20)^ found that 87% of patients undergoing thoracic or abdominal cancer surgery died, whereas 56% of patients with a prolonged hospital stay had some type of complication. Davies et al.^(18)^ validated the POMS using 362 patients undergoing abdominal surgery, of whom 75% underwent gastrointestinal surgery, and found that the occurrence of complications significantly prolonged hospital stays.

The negative effects of postoperative complications are not necessarily immediate or short term. Moonesinghe et al.^(21)^ used the POMS to evaluate the postoperative complications following different surgical specialties and found that these complications were strongly associated with mortality at three years. Similarly, the time complication was associated with longer hospital stays.

An understanding of the clinical implications of postoperative complications requires an understanding of the effect of each specific type of complication. For example, Fleisher and Linde-Zwirble found that lung and cardiovascular complications were present in 20.8% and 2.9% of patients undergoing gastrointestinal surgery, respectively; however, these complications accounted for 64% and 4% of all hospital deaths.^(19)^ Although we found fewer lung complications and more cardiovascular complications, their effects on hospital mortality were high for both. This finding is most likely because of the distribution of each particular type of complication within each group in addition to the features of the sample and the protocols for managing the complications at each center.

The results concerning gastrointestinal complications were similar to those reported by other authors.^(22,23)^ In addition, the Kidney Disease: Improving Global Outcomes (KDIGO) Acute Kidney Injury Work Group recognized surgery as a major risk factor for acute renal failure,^(24)^ whereas (consistent with our study) Hoste et al.^(25)^ showed that acute renal failure increased hospital mortality among patients undergoing surgery, especially those with infections.

Infections represent a significant aspect in the evaluation of postoperative clinical outcomes, primarily because of the epidemiological implications involved. Avritscher et al.^(26)^ reported an infection rate among patients receiving thoracic or gastrointestinal cancer surgeries similar to that found in our study; likewise, they showed that infections were significantly associated with increased mortality and longer hospital stays. These results coincide with those for patients undergoing surgery who were admitted to surgical ICUs.^(27,28)^ Adherence to postoperative infection prevention programs can help reduce the incidence of infection and improve clinical outcomes.^(29)^

Postoperative morbidity and mortality rates often vary across different hospitals and healthcare systems, including within the context of critical care.^(30,31)^ Some complications are difficult to avoid, particularly among high-risk patients with multiple comorbidities. However, the frequencies of complications and mortality can be reduced by improving the structure and process of healthcare. The implementation of therapeutic strategies such as goal-directed fluid therapy,^(32,33)^ enhanced recovery after surgery programs,^(34,35)^ and expanding the provision of critical care services enables a greater number of high-risk patients to be managed with intensive monitoring and treatment. These measures can help to improve postoperative clinical outcomes.

## CONCLUSIONS

The current research shows that the lung, cardiovascular, gastrointestinal, infectious disease, and renal complications following surgeries for thorax or digestive tract cancers are associated with increased hospital mortality. Similarly, the occurrence of any postoperative complication increases the likelihood of remaining hospitalized. The systematic use of complications to indicate postoperative clinical outcomes is suggested. The current results suggest the need for additional studies aimed at implementing changes to the structure and processes related to healthcare to reduce postoperative morbidity and mortality. These changes might be feasible by conducting a clinical trial showing that the execution of a prevention protocol reduces the incidence of complications and mortality.
